# K_ATP_ channels and NO dilate redundantly intramuscular arterioles during electrical stimulation of the skeletal muscle in mice

**DOI:** 10.1007/s00424-021-02607-1

**Published:** 2021-08-13

**Authors:** Simon Schemke, Cor de Wit

**Affiliations:** 1 Institut für Physiologie, Universität zu Lübeck, Ratzeburger Allee 160, 23562 Lübeck, Germany; 2 Deutsches Zentrum für Herz-Kreislauf-Forschung (DZHK) E.V. (German Center for Cardiovascular Research), partner site Hamburg/Kiel/Lübeck, Lübeck, Germany

**Keywords:** Adenosine, K_ATP_ channels, Glibenclamide, Endothelial autacoids, Active hyperemia

## Abstract

Functional hyperemia is fundamental to provide enhanced oxygen delivery during exercise in skeletal muscle. Different mechanisms are suggested to contribute, mediators from skeletal muscle, transmitter spillover from the neuromuscular synapse as well as endothelium-related dilators. We hypothesized that redundant mechanisms that invoke adenosine, endothelial autacoids, and K_ATP_ channels mediate the dilation of intramuscular arterioles in mice. Arterioles (maximal diameter: 20–42 µm, *n* = 65) were studied in the cremaster by intravital microscopy during electrical stimulation of the motor nerve to induce twitch or tetanic skeletal muscle contractions (10 or 100 Hz). Stimulation for 1–60 s dilated arterioles rapidly up to 65% of dilator capacity. Blockade of nicotinergic receptors blocked muscle contraction and arteriolar dilation. Exclusive blockade of adenosine receptors (1,3-dipropyl-8-(p-sulfophenyl)xanthine) or of NO and prostaglandins (nitro-*L*-arginine and indomethacin, LN + Indo) exerted only a minor attenuation. Combination of these blockers, however, reduced the dilation by roughly one-third during longer stimulation periods (> 1 s at 100 Hz). Blockade of K_ATP_ channels (glibenclamide) which strongly reduced adenosine-induced dilation reduced responses upon electrical stimulation only moderately. The attenuation was strongly enhanced if glibenclamide was combined with LN + Indo and even observed during brief stimulation. LN was more efficient than indomethacin to abrogate dilations if combined with glibenclamide. Arteriolar dilations induced by electrical stimulation of motor nerves require muscular contractions and are not elicited by acetylcholine spillover from neuromuscular synapses. The dilations are mediated by redundant mechanisms, mainly activation of K_ATP_ channels and release of NO. The contribution of K^+^ channels and hyperpolarization sets the stage for ascending dilations that are crucial for a coordinated response in the network.

## Introduction

Functional or active hyperemia is a fundamental mechanism to provide adequate oxygen delivery during enhanced workloads and is of specific importance in the heart and skeletal muscle. In these organs, oxygen demand may increase substantially which can be only met by approximate proportional increases in blood flow [9, 22, 31, 43]. To allow such large increases in blood flow, vascular resistance has to decrease considerably which can only be brought about by substantial dilation in arterioles and small arteries. Over the years, many different mediators and mechanisms have been suggested to contribute to the dilation in skeletal muscle in response to exercise. In various studies, mediators released from the actively contracting skeletal muscle fibers (e.g., adenosine, potassium, lactate) have been implicated to contribute to the dilation [24, 33, 40, 44]. In addition, acetylcholine spillover from the neuromuscular synapse was suggested to act decisively in the coupling between muscular contraction and vascular dilation [47]. The mechanical deformation associated with muscular contraction acting on the arterioles residing in the skeletal muscle itself may be an important signal to stimulate the endothelium to release dilator mediators such as nitric oxide (NO) and prostaglandins [19], and may also activate the endothelium-dependent hyperpolarization (EDH) mechanism to relax the smooth muscle [2].

The role of NO has been examined extensively in different species early after the discovery of the crucial role of endothelial-derived NO in arteriolar dilation, and many demonstrated a contribution of NO in functional hyperemia [15, 23, 41, 42]. This NO release may be produced by enhanced shear stress acting on endothelial cells [34] or by mediators released from the active skeletal muscle. In addition, neuronal NO synthase (nNOS) was shown to be activated during twitching in skeletal muscle, and NO derived from nNOS contributed to arteriolar dilation during hyperemia [21]. Interestingly, NO release from the endothelium was also elicited by pure passive mechanical compression of rat hearts leading to enhanced coronary flow as well as by deformation of isolated arterioles resulting in NO-mediated dilation [46]. However, this view was also challenged in numerous studies in which NO accounted only for a minor part (if any) of the dilation in active hyperemia [4]. In humans, NOS inhibition attenuated blood flow during exercise only in combination with blockade of prostaglandin synthesis indicating a substantial redundancy between these two systems [14], whereas EDH cannot compensate their impairment [28].

Adenosine is a powerful vasodilator that is generated by the ecto-5′nucleosidase from adenosinmonophosphate (AMP) being released from skeletal muscle during exercise at reduced oxygen tensions [24]. Numerous studies demonstrated already more than 20 years ago that adenosine receptor antagonists reduced exercise hyperemia in different species [35], 36. In accordance with this view, strictly localized contractions of a small number of muscle fibers in the hamster cremaster induced a dilation that was also partially reduced by a blockade of adenosine receptors [30]. This sophisticated setup with restricted local fiber stimulation allowed to examine a possible transfer of a dilator signal along the arteriolar tree, the so-called conducted dilation that requires the coupling of the vascular cells to transmit a dilatory signal [6]. Interestingly, K_ATP_ channels are also involved in these responses, and their activity was required at the local as well as at the remote arteriole to initiate the dilation. Because the blockade of K_ATP_ channels did not block dilations of adenosine, the authors concluded that adenosine and K_ATP_ channels are separate effectors that both contribute independently to dilation in response to muscle contraction [30].

In most studies, hyperemia was performed in intact organs, and blood flow into the skeletal muscle during exercise was measured by different means, thereby assessing the change of conductance in the overall vascular bed. However, the mechanisms leading to dilation may vary between different vessel sizes and generations, and detailed information on the site of action of distinct mechanisms along the vascular tree remains unknown. Moreover, considerable variety between species is well known with regard to mechanisms of dilation [25]. For example, arterioles in the murine microcirculation rely heavily on the EDH mechanism and K_Ca_ channels if the endothelium is stimulated with acetylcholine [37, 45]. Therefore, we examined arteriolar dilation in the murine cremaster muscle microcirculation by intravital microscopy and observed intramuscular arterioles before and during electrical stimulation of this skeletal muscle. We hypothesized that endothelial-derived NO and prostaglandins as well as adenosine contribute to the dilation in these arterioles during muscular exercise. To test this hypothesis, exercise-induced dilations were studied before and after blockade of NO synthase, cyclooxygenase, and/or adenosine receptor blockade. Adenosine is reported to exert its dilatory effect upon receptor stimulation via activation of K_ATP_ channels [16, 18, 32, 38], while others suggested that its effect is independent of such channels [10, 30]. Therefore, we also examined the role of K_ATP_ channels in these responses.

## Material and methods

### Animals

Animal care and experiments were in accordance with the German Animal Welfare Act and approved by local authorities (Ministerium für Landwirtschaft, Umwelt und ländliche Räume des Landes Schleswig–Holstein). In total, 65 male mice with a C57BL/6 genetic background were studied at an age between 3 and 6 months.

### Intravital microscopy of the microcirculation

Mice were anesthetized with intraperitoneal injection of fentanyl (0.05 mg/kg), midazolam (5 mg/kg), and medetomidin (0.5 mg/kg) in a volume of 13 mL/kg. A jugular vein catheter allowed subsequent continuous infusion of the anesthetic drugs (at 5 mL/kg/h and 0.02, 2.2, 0.22 mg/kg/h of fentanyl, midazolam, and medetomidin). A small tube was inserted into the trachea to secure the airway and to ventilate the animal during the experiment with a tidal volume of 200 µl at a rate of 150/min (MicroVent Mouse Ventilator, Hugo Sachs Elektronik, Hugstetten, Germany). The cremaster muscle was prepared as described [17, 37] superfused with a tempered (34 °C) buffer containing (in mmol/L) the following: 118.4 NaCl, 20 NaHCO_3_, 3.8 KCl, 2.5 CaCl_2_, 1.2 KH_2_PO_4_, and 1.2 MgSO_4_. The buffered (pH 7.4) saline solution was gassed with 5% CO_2_ and 95% N_2_ resulting in a pO_2_ of about 30 mmHg on the cremaster muscle due to contamination with ambient air. All drugs were applied in the superfusion. In each mouse, one arteriole was observed through a 40-fold objective using a video camera-equipped microscope (Axioscope FS, Zeiss, Germany). Images were recorded on videotape to allow later measurement of the inner arteriolar diameter with an application written in LabVIEW8.0 (National Instruments, Austin, TX, USA). This application projected over digitized images a rectangle which boundaries marked the inner arteriolar diameter. For diameter measurements, the user adjusted the boundaries of this rectangle by simple key strokes during replay of the microscopic images at reduced speed. Values were sampled at a frequency of 2 Hz.

### Experimental procedure

After equilibration for 30 min, vascular reactivity of the selected arteriole was assessed by applying acetylcholine (ACh) and adenosine (10 µmol/L) before studying muscular contraction induced responses. Skeletal muscle contractions were initiated by train pulses at 10 or 100 Hz for 0.5 s every 3 s of about 2.5 V (WPI Anapulse Stimulator 302-T, WPI, Sarasota, USA) delivered through a microelectrode (platinum wire, 25 μm) placed in the vicinity of the motor nerve at the base of the cremaster muscle. Train stimulations were continued for 1, 15, 30, or 60 s causing clearly visible skeletal muscle contraction every third second. During the 0.5-s stimulation period, muscle contractions were interrupted by relaxations at 10 Hz stimulation. Although these relaxations may not have been complete and the contractions, in fact, may have partially overlapped, we refer to them as twitch contractions. In contrast, stimulation at 100 Hz induced tetanic skeletal muscle contractions. Arteriolar diameter was monitored before and after stimulation as well as between contractions during the 2.5-s interval of stimulation break. After stimulation, vessels were allowed to recover tone (within 3 to 8 min) before studying the next stimulation period. This experimental protocol was repeated in different subgroups of experiments either after inhibition of NO synthase and cyclooxygenase by nitro-*L*-arginine and indomethacin (LN, Indo; 30 and 3 µM) blocking efficiently NO synthase and cyclooxygenase [48, 49], in the presence of the non-selective adenosine receptor blocker 1,3-dipropyl-8-(p-sulfophenyl)xanthine (10 µM, DPSX), the K_ATP_ channel blocker glibenclamide (10 µmol/L, Glib) which efficiently blocks these channels in this preparation [7], or a combination of these treatments. In a small group of animals, the nicotinic receptor blocker pancuronium (1 µmol/L) was applied as treatment. Importantly, the protocol in each experimental subgroup was initially performed in untreated preparations which served as the respective control to generate paired experimental data sets. At the end of each experiment, the maximal diameter of the arterioles was determined by combined superfusion of ACh, adenosine, and sodium nitroprusside (each 30 µmol/L). Animals were then sacrificed by an overdose of pentobarbital (2.5 g/kg) applied intravenously.

### Data analysis

Vascular tone is given as percentage of maximal diameter. Internal diameters are measured and changes normalized to the dilator capacity according to the formula:

$$\mathrm{\%\;of\;dilator\;capacity}=(\mathrm{D}_{\mathrm{Stim}} - \mathrm{D}_{\mathrm{Con}}) / (\mathrm{D}_{\mathrm{Max}} - \mathrm{D}_{\mathrm{Con}})\times 100$$

with *D*_Stim_ being the diameter during stimulation, *D*_Con_ the control diameter before stimulation, and *D*_Max_ the maximal diameter measured for the arteriole during the experiment which included the combined superfusion of different dilators at supramaximal concentrations. Statistical analysis was performed using STATA (Stata Corporation TX, USA). Data are presented as a mean ± standard error of mean (mean ± SEM). Time series measurements (diameter before and after electrical stimulation) were analyzed by repeated measures ANOVA followed by post hoc analysis of the means corrected according to Bonferroni. Paired samples (treatment vs control) were analyzed by paired *t*-test. Differences were considered significant at an error probability of *P* < 0.05.

## Results

### Active hyperemia in the skeletal muscle microcirculation

A total of 65 arterioles were studied in 65 mice. Arterioles under study were second (2A) and third-order arterioles (3A) with a maximal diameter that ranged from 20 to 42 µm (mean of 29.1 ± 0.7 µm). Arteriolar resting diameter amounted to 8.1 ± 0.5 µm, which represented a resting tone (resting normalized to maximal diameter) of 27.3 ± 1.1% with a range from 12 to 57%. Acetylcholine induced in these arterioles a concentration-dependent dilation (1 µM, 27.6 ± 3.2%; 10 µM, 68.7 ± 2.6%; *P* < 0.05) indicating functionally intact endothelium-dependent dilations. Likewise, adenosine induced concentration-dependent dilations (1 µM, 14.0 ± 1.9%; 10 µM, 55.2 ± 2.9%; *P* < 0.05).

**Fig. 1:**
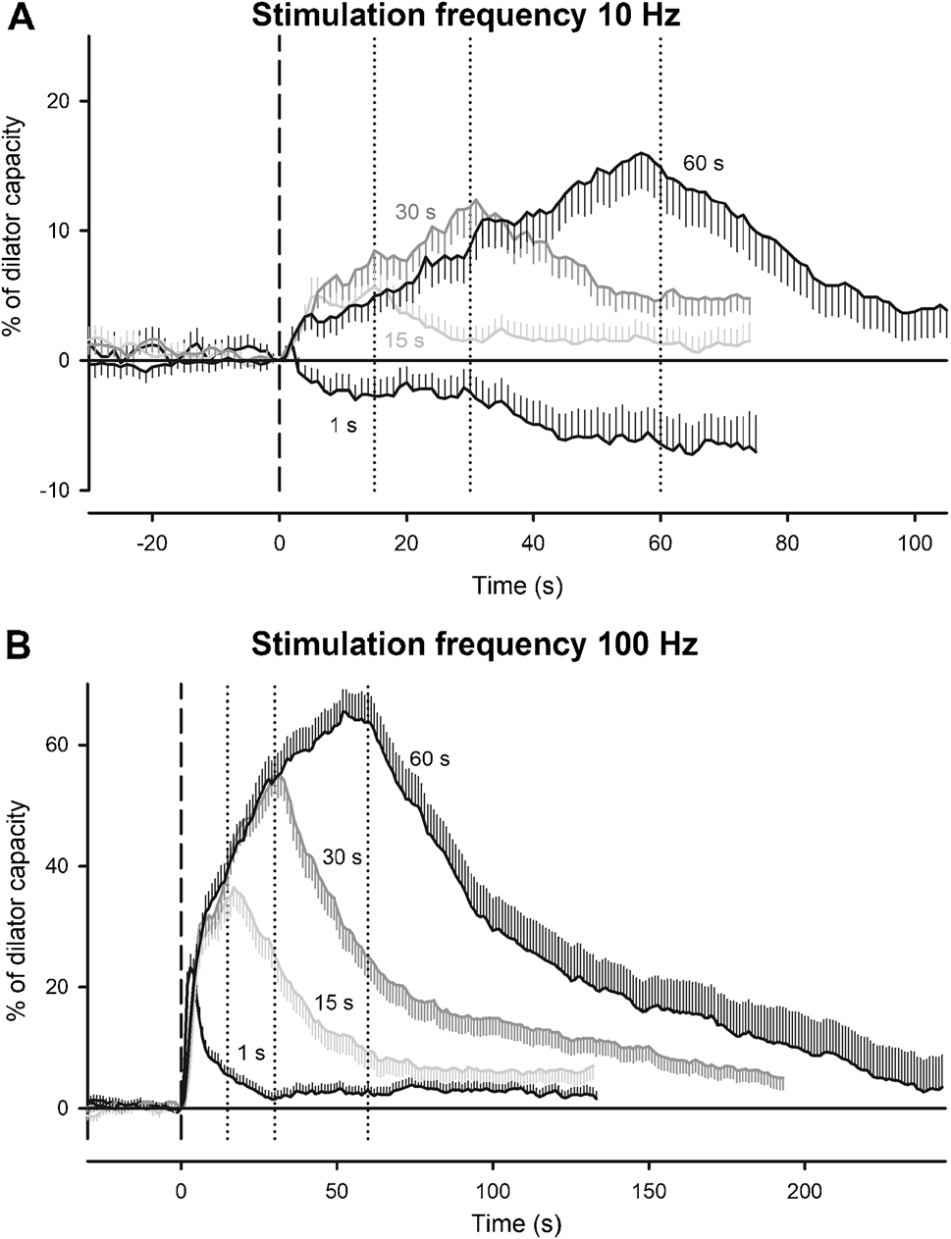
Skeletal muscle contraction induces an arteriolar dilation which is dependent on work intensity. Stimulation of the motor nerve in the cremaster muscle induces visible skeletal muscle contraction and arteriolar dilation. In order to enable the visibility of the arterioles under study, 0.5 s of stimulation was followed by a break lasting for 2.5 s. The dilation was substantially larger if the skeletal muscle was stimulated with a tetanic frequency (100 Hz, B) compared to a twitch frequency (10 Hz, A). The amplitude of the dilation increased with stimulation duration, and the slope of the dilation was comparable between different stimulation periods. Interestingly, the slope of the dilatory curves in response to 100 Hz stimulation was initially steeper compared to later time points. Dilations is expressed as percent of dilator capacity, *n* = 65 arterioles in 65 mice except for 1-s stimulation with 10 Hz (*n* = 45). Dashed line indicates start of stimulation at 0 s, the duration of the stimulation is given for each curve as text, and dotted lines indicate the end of stimulation (15, 30, and 60 s, respectively). Statistical comparison for different time points is given in text

Upon stimulation of the motor nerve, the skeletal muscle visibly contracted, and arterioles dilated quickly. The amplitude of the dilation was dependent on stimulation frequency and duration of the stimulation (Fig. 1). At a stimulation frequency of 10 Hz, which induces twitch contractions, significant dilations were observed at stimulation durations of 15, 30, and 60 s, whereas 1-s stimulation did not significantly dilate the arterioles. Dilations induced at a frequency of 100 Hz (inducing tetanic contractions) were significantly larger at all stimulation durations compared to 10 Hz. Specific time points were analyzed, firstly, during stimulation (at 50% of total stimulation time, e.g., at 7.5, 15, and 30 s for 15, 30, and 60-s stimulation duration: 15 s 4.5 ± 1.2 vs 28.1 ± 2.5%; 30 s 7.6 ± 1.5 vs 38.2 ± 2.8%; 60 s 9.0 ± 1.8 vs 54.2 ± 3.7%; 10 vs 100 Hz, respectively, all *P* < 0.05), secondly, at the end of the stimulation (15 s 5.5 ± 1.4 vs 34.3 ± 3.1%; 30 s 11.5 ± 2.0 vs 54.3 ± 3.0%; 60 s 15.3 ± 2.7 vs 63.8 ± 4.4%; 10 vs 100 Hz: all *P* < 0.05), and thirdly after the stimulation (50% of the stimulation time after the end of stimulation, e.g., at 7.5, 15, and 30 s after the end of stimulation for 15, 30, and 60-s stimulation duration: 15 s 2.9 ± 0.8 vs 32.3 ± 3.3%; 30 s 7.4 ± 1.5 vs 37.5 ± 3.8%; 60 s 6.0 ± 2.3 vs 37.1 ± 5.0%; 10 vs 100 Hz: all *P* < 0.05). Because values during stimulation are not available for the shortest stimulation period (1 s) and the dilation of the arteriole takes a minimal time to achieve its maximum (Fig. 1), time points for analysis are different, but chosen in analogy. Values during stimulation are represented by the diameter immediately after stimulation. Accordingly, values for the end of stimulation are given as diameters observed 2 s after ending, and values after stimulation are given as diameters obtained 4 s after ending the stimulation. A significant dilation was observed even at the shortest duration of 1 s at 100 Hz (during, 5.7 ± 1.0%; end, 21.7 ± 2.0; after, 16.9 ± 2.2%; all values *P* < 0.05 vs diameter before stimulation), but not at 10 Hz (end: -0.9 ± 1.2%).

The slope of the ensuing dilation during stimulation (diameter change in µm) was calculated for every 15-s interval until 45 s after start of the stimulation. For the same stimulation frequency, the slope was similar for different stimulation durations (data not shown). However, the slope was significantly steeper for the higher stimulation frequency during the first 30 s (0 to 15 s, 1.3 ± 0.2 vs 8.0 ± 0.4; 15 to 30 s, 0.7 ± 0.2 vs 3.2 ± 0.2 µm/15 s, 10 vs 100 Hz, respectively, both *P* < 0.05). For the last 15 s interval, a tendency was observed (30 to 45 s: 0.8 ± 0.2 vs 1.5 ± 0.3 µm/15 s, 10 vs 100 Hz, *P* = 0.07). The slope of the dilation flattened with longer stimulation durations particularly at 100 Hz stimulation (from initially 8.0 ± 0.4 to 3.2 ± 0.2 µm/15 s, *P* < 0.05, to finally 1.5 ± 0.3 µm/15 s for the last 15-s interval, *P* < 0.05 vs the second interval from 15 to 30 s). Such a decrease in the slope was found during 10 Hz stimulation only from the first to the second 15-s interval (1.3 ± 0.2 vs 0.7 ± 0.2, *P* < 0.05). Noteworthy, any further progress of the dilation was immediately (within seconds) interrupted after cessation of the electrical stimulation (Fig. 1).

### Skeletal muscle contraction is required to induce arteriolar dilation

Nicotinergic neurotransmission was blocked by application of pancuronium (Panc, 1 µM), which did not change arteriolar diameters (Con, 10.3 ± 1.3 µm; Panc, 10.3 ± 1.0 µm, *n* = 12). In this subgroup, stimulation at 100 Hz for 1 s induced a dilation of 8.6 ± 3.7% and stimulation for 30 s a dilation of 33.1 ± 8.0% (both *P* < 0.05) before treatment. In the presence of Panc, skeletal muscle contractions upon motor nerve stimulation were no longer visible and arteriolar diameter remained unchanged at the end of the stimulation period (1 s: 0.6 ± 1.6%, 30 s: 2.7 ± 4.4%; both *P* = ns; Table 1). However, ACh-induced dilations remained unaltered (1 µM, 13.5 ± 3.2 vs 17.2 ± 8.0%; 10 µM, 62.3 ± 6.3 vs 56.5 ± 9.0%, Panc vs control, respectively).

**Table 1:** Arteriolar diameters before, during, at the end and after electrical stimulation of the skeletal muscle at 100 Hz for different stimulation durations before and after treatment in the different subgroups. All values are given as mean ± SEM in µm; *n* indicates number of arterioles; maximum is the maximal arteriolar diameter observed during the experiment including values obtained during superfusion of different dilators at supramaximal concentrations, *P < 0.05 vs diameter before stimulation (repeated measures ANOVA followed by post hoc analysis of the means)

Treatment	Duration	Diameter (µm)				
		Before	During	End	After	Maximum
Nicotinergic receptor blockade: pancuronium (Panc, 1 µM, *n* = 12)						
Con	1 s 30 s	9.5 ± 1.4 10.5 ± 1.7	9.6 ± 1.3 14.6 ± 2.2^*^	11.5 ± 0.9^*^ 17.6 ± 2.5^*^	11.8 ± 1.1 14.6 ± 2.5^*^	31.2 ± 1.5 31.2 ± 1.5
Panc	1 s 30 s	10.4 ± 1.3 10.0 ± 1.3	10.4 ± 1.2 11.1 ± 1.5	10.6 ± 1.2 10.8 ± 1.7	10.5 ± 1.1 9.8 ± 1.4	31.2 ± 1.5 31.2 ± 1.5
Adenosine receptor blockade: 1,3-dipropyl-8-(p-sulfophenyl)xanthine (DPSX, 10 µM, *n* = 7)						
Con	1 s 15 s 30 s 60 s	9.0 ± 1.3 9.9 ± 1.5 10.0 ± 1.2 9.8 ± 1.2	9.3 ± 1.1 14.1 ± 1.8^*^ 17.5 ± 2.7^*^ 23.8 ± 2.7^*^	12.9 ± 1.6^*^ 16.7 ± 1.6^*^ 21.3 ± 2.2^*^ 26.2 ± 2.8^*^	14.5 ± 2.1^*^ 14.1 ± 2.1 16.5 ± 3.2 17.9 ± 3.2^*^	29.7 ± 2.5 29.7 ± 2.5 29.7 ± 2.5 29.7 ± 2.5
DPSX	1 s 15 s 30 s 60 s	8.1 ± 1.0 8.5 ± 0.9 8.2 ± 0.8 7.6 ± 1.0	8.7 ± 1.2 15.3 ± 1.9^*^ 15.9 ± 1.3^*^ 20.6 ± 2.1^*^	12.2 ± 1.8^*^ 16.3 ± 1.7^*^ 20.4 ± 1.8^*^ 21.6 ± 2.2^*^	14.8 ± 2.5^*^ 13.2 ± 1.7^*^ 14.9 ± 1.5^*^ 13.2 ± 1.6^*^	29.7 ± 2.5 29.7 ± 2.5 29.7 ± 2.5 29.7 ± 2.5
Inhibition of endothelial autacoids: nitro-*L*-arginine and indomethacin (LN + Indo; 30 and 3 µM, *n* = 8)						
Con	1 s 15 s 30 s 60 s	8.9 ± 0.9 9.1 ± 1.0 9.8 ± 1.2 10.1 ± 1.7	10.0 ± 0.8 14.7 ± 1.8^*^ 17.0 ± 2.2 ^*^ 19.7 ± 3.1^*^	11.7 ± 1.2^*^ 16.1 ± 1.8^*^ 20.3 ± 2.6^*^ 22.3 ± 3.4^*^	11.3 ± 1.6 15.6 ± 1.8^*^ 18.9 ± 2.7^*^ 19.0 ± 2.8^*^	30.3 ± 2.5 30.3 ± 2.5 30.3 ± 2.5 30.3 ± 2.5
LN + Indo	1 s 15 s 30 s 60 s	5.9 ± 0.9 6.0 ± 0.6 6.0 ± 1.0 6.6 ± 0.9	6.0 ± 0.9 10.5 ± 1.8^*^ 13.1 ± 1.8^*^ 19.3 ± 2.4^*^	7.9 ± 1.1^*^ 14.7 ± 2.4^*^ 19.2 ± 2.5^*^ 23.0 ± 3.2^*^	8.3 ± 1.3^*^ 12.7 ± 2.1^*^ 18.7 ± 2.7^*^ 18.3 ± 2.6^*^	30.3 ± 2.5 30.3 ± 2.5 30.3 ± 2.5 30.3 ± 2.5
Blockade of adenosine receptors and endothelial autacoid synthesis: LN + Indo + DPSX (*n* = 24)						
Con	1 s 15 s 30 s 60 s	7.7 ± 0.6 7.7 ± 0.7 8.0 ± 0.7 8.4 ± 0.9	8.9 ± 0.5^*^ 13.9 ± 1.0^*^ 16.4 ± 1.2^*^ 20.9 ± 1.4^*^	12.5 ± 0.6^*^ 15.5 ± 0.9^*^ 20.3 ± 1.2^*^ 23.3 ± 1.5^*^	11.4 ± 1.0^*^ 14.9 ± 0.9^*^ 17.1 ± 1.4^*^ 18.0 ± 1.5^*^	28.1 ± 1.3 28.1 ± 1.3 28.1 ± 1.3 28.1 ± 1.3
LN + Indo + DPSX	1 s 15 s 30 s 60 s	3.6 ± 0.5 4.2 ± 0.6 4.7 ± 0.6 4.5 ± 0.6	4.5 ± 0.6^*^ 11.1 ± 1.2^*^ 11.1 ± 1.1^*^ 15.2 ± 1.6^*^	8.3 ± 1.0^*^ 11.3 ± 1.0^*^ 16.3 ± 1.4^*^ 17.3 ± 1.7^*^	8.8 ± 1.1^*^ 10.6 ± 1.0^*^ 13.4 ± 1.4^*^ 11.9 ± 1.6^*^	28.1 ± 1.3 28.1 ± 1.3 28.1 ± 1.3 28.1 ± 1.3
K_ATP_ channel blockade: glibenclamide (Glib, 10 µM, *n* = 15)						
Con	1 s 15 s 30 s 60 s	6.6 ± 0.6 6.6 ± 0.7 6.6 ± 0.7 6.8 ± 0.7	8.5 ± 0.6 15.1 ± 1.4^*^ 18.2 ± 1.3^*^ 22.5 ± 1.7^*^	12.9 ± 0.9^*^ 16.5 ± 1.4^*^ 21.7 ± 1.3^*^ 24.3 ± 1.3^*^	12.1 ± 1.4^*^ 15.7 ± 1.8^*^ 17.7 ± 2.1^*^ 18.2 ± 2.1^*^	29.1 ± 1.3 29.1 ± 1.3 29.1 ± 1.3 29.1 ± 1.3
Glib	1 s 15 s 30 s 60 s	3.0 ± 0.4 3.8 ± 0.6 5.1 ± 0.8 6.2 ± 0.9	4.4 ± 0.4^*^ 13.3 ± 1.4^*^ 12.8 ± 1.5^*^ 17.7 ± 1.4^*^	9.4 ± 1.1^*^ 13.0 ± 1.6^*^ 17.7 ± 1.4^*^ 21.6 ± 1.5^*^	10.6 ± 1.3^*^ 10.0 ± 1.8^*^ 13.7 ± 1.9^*^ 15.0 ± 1.7^*^	29.1 ± 1.3 29.1 ± 1.3 29.1 ± 1.3 29.1 ± 1.3
Blockade of K_ATP_ channels and endothelial autacoid synthesis: Glib + LN + Indo (*n* = 5)						
Con	1 s 15 s 30 s 60 s	5.2 ± 1.1 4.7 ± 1.3 5.6 ± 1.3 4.9 ± 1.1	8.8 ± 1.4 14.7 ± 2.7^*^ 17.1 ± 3.5^*^ 22.1 ± 4.1^*^	14.1 ± 1.4^*^ 15.2 ± 2.7^*^ 20.9 ± 2.8^*^ 24.0 ± 3.0^*^	10.0 ± 2.2^*^ 15.6 ± 3.9^*^ 19.2 ± 4.1^*^ 19.1 ± 4.3^*^	27.7 ± 2.8 27.7 ± 2.8 27.7 ± 2.8 27.7 ± 2.8
Glib + LN + Indo	1 s 15 s 30 s 60 s	1.2 ± 0.5 1.5 ± 0.7 1.3 ± 0.5 1.1 ± 0.3	4.0 ± 2.5 5.9 ± 2.8 6.1 ± 2.6 2.8 ± 1.0	6.2 ± 3.1 4.1 ± 1.9 3.7 ± 1.4 6.3 ± 2.8	6.6 ± 2.5 3.0 ± 1.3 3.4 ± 0.9 3.6 ± 1.3	27.7 ± 2.8 27.7 ± 2.8 27.7 ± 2.8 27.7 ± 2.8
Blockade of K_ATP_ channels and NO synthase: Glib + LN (*n* = 5)						
Con	1 s 15 s 30 s 60 s	6.8 ± 0.4 6.3 ± 1.0 5.0 ± 0.5 6.4 ± 0.6	8.3 ± 0.7^*^ 11.6 ± 1.5 17.1 ± 1.6^*^ 21.9 ± 1.5^*^	11.2 ± 1.3^*^ 15.2 ± 2.3^*^ 20.0 ± 2.1^*^ 23.5 ± 1.5^*^	9.6 ± 1.2 15.6 ± 2.8^*^ 15.7 ± 3.5^*^ 18.6 ± 2.6^*^	28.0 ± 1.9 28.0 ± 1.9 28.0 ± 1.9 28.0 ± 1.9
Glib + LN	1 s 15 s 30 s 60 s	1.6 ± 0.4 1.5 ± 0.5 1.6 ± 0.4 2.3 ± 0.5	1.6 ± 0.4 7.3 ± 2.9 5.9 ± 1.6 10.2 ± 3.2	2.5 ± 1.2 5.1 ± 1.6 7.7 ± 2.7 12.2 ± 3.7^*^	4.0 ± 2.8 3.3 ± 1.0 5.7 ± 1.9 8.0 ± 2.7	28.0 ± 1.9 28.0 ± 1.9 28.0 ± 1.9 28.0 ± 1.9
Blockade of K_ATP_ channels and cyclooxygenase: Glib + Indo (*n* = 5)						
Con	1 s 15 s 30 s 60 s	7.7 ± 1.0 8.7 ± 0.6 9.3 ± 0.7 9.2 ± 0.9	8.4 ± 0.8 19.1 ± 1.6^*^ 20.3 ± 1.2^*^ 23.6 ± 3.2^*^	13.2 ± 1.7 19.2 ± 2.2^*^ 24.3 ± 1.9^*^ 25.4 ± 2.3^*^	16.8 ± 2.2^*^ 15.8 ± 3.3 18.3 ± 4.2 16.9 ± 4.7	31.6 ± 1.6 31.6 ± 1.6 31.6 ± 1.6 31.6 ± 1.6
Glib + Indo	1 s 15 s 30 s 60 s	2.7 ± 1.1 3.6 ± 1.3 3.7 ± 1.7 5.8 ± 0.9	3.7 ± 1.3 12.3 ± 3.4^*^ 16.4 ± 2.5^*^ 16.3 ± 2.8^*^	7.1 ± 2.7 15.4 ± 2.8^*^ 17.8 ± 3.4^*^ 19.4 ± 3.9^*^	9.2 ± 3.1 12.2 ± 1.3^*^ 13.1 ± 2.6^*^ 14.8 ± 4.3	31.6 ± 1.6 31.6 ± 1.6 31.6 ± 1.6 31.6 ± 1.6

### Active hyperemia was only reduced by the combined blockade of adenosine receptors and of NO synthase and cyclooxygenase

**Fig. 2:**
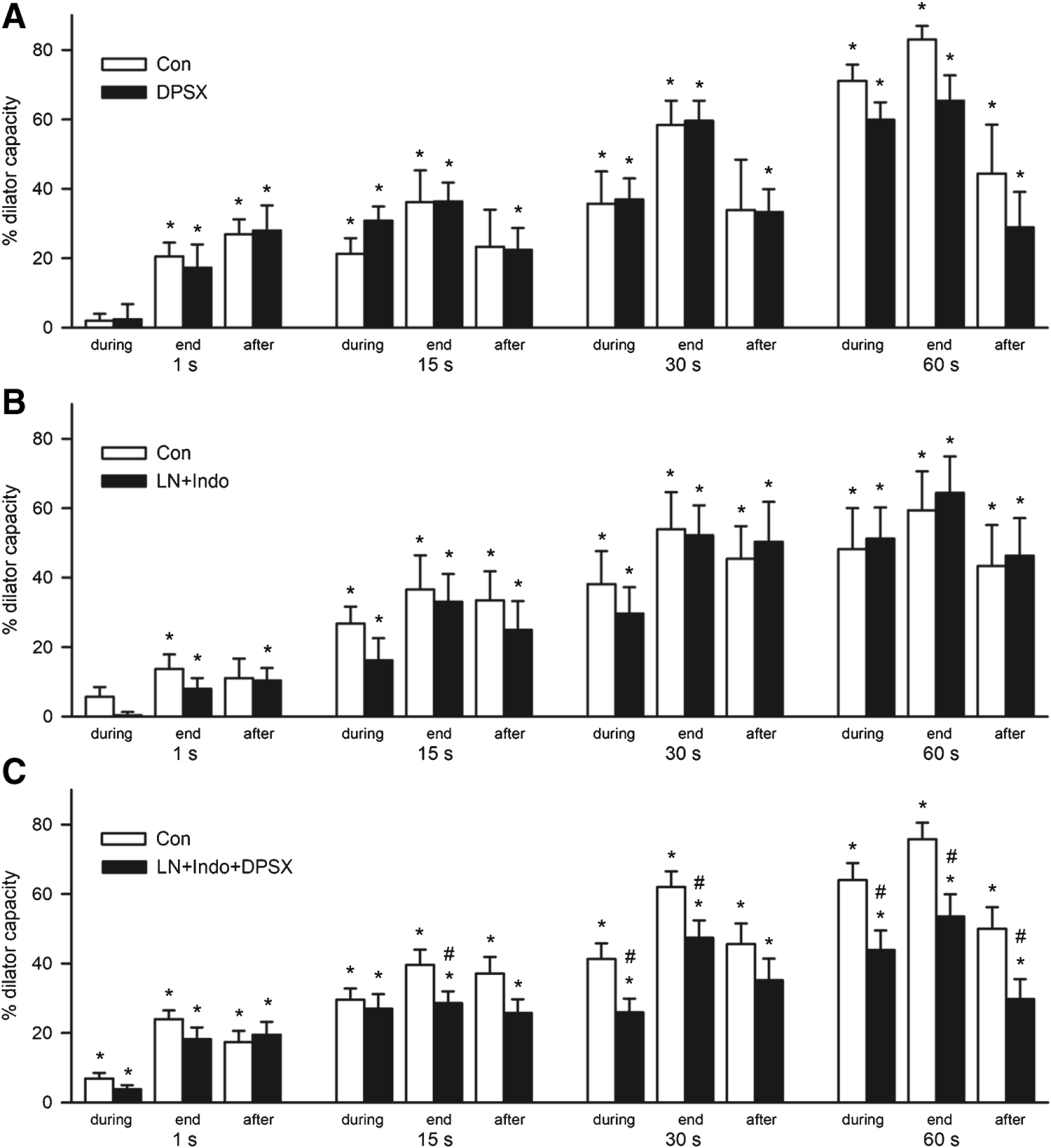
Blockade of adenosine receptors and inhibition of the synthesis of the endothelial autacoids NO and prostaglandins reduced active hyperemia only in combination. Arteriolar dilations during, at the end of, and after stimulation of skeletal muscle contraction at 100 Hz are depicted for the different stimulation periods. Treatments are shown in black bars; the respective control values obtained in the exact same arterioles are depicted in white bars. **A** Application of the non-selective adenosine receptor blocker 1,3-dipropyl-8-(p-sulfophenyl)xanthine (10 µM, DPSX, *n* = 7) did not change the arteriolar dilations. **B** The inhibition of NO synthase and cyclooxygenase (LN, Indo; 30 and 3 µM, *n* = 8) did also not affected active hyperemia. **C** A combination of these treatments (*n* = 24) reduced arteriolar dilations at the end of the stimulation for 15, 30, and 60 s and also during stimulation at longer stimulation periods. Dilation is given as percent of dilator capacity, *P < 0.05 vs diameter before stimulation; #*P* < 0.05 vs dilation of respective control; diameter values are given in Table 1. Specific time points are depicted: (1) During stimulation is the time point at 50% of total stimulation time (e.g., at 7.5, 15, and 30 s after the start of the stimulation for 15, 30, and 60-s stimulation periods, respectively), (2) end is at the end of stimulation, and (3) after stimulation is time point at 50% of the stimulation time after the end of stimulation (e.g., at 7.5, 15, and 30 s after the end of stimulation for 15, 30, and 60 s stimulation periods, respectively). For the shortest stimulation period (1 s), during stimulation, values are not available. Therefore, time points are chosen in analogy: (1) During stimulation is represented by the diameter value immediately after stimulation (1 s), (2) end of the stimulation is 2 s after the end of stimulation, and (3) after stimulation is the value obtained 4 s after the end of stimulation

The role of adenosine and/or the endothelial autacoids NO and prostaglandins were studied by application of the non-selective adenosine receptor blocker 1,3-dipropyl-8-(p-sulfophenyl)xanthine (10 µM, DPSX) or by inhibition of the NO synthase and cyclooxygenase (LN, Indo; 30 and 3 µM) and the combination of these treatments in different subgroups. DPSX did not change resting arteriolar diameters (Con, 9.5 ± 1.3 µm; DPSX, 8.4 ± 1.0 µm, *n* = 7). However, the dilation induced by adenosine (10 µM) was nearly abrogated after DPSX (from 51.8 ± 12.9 to 7.3 ± 2.3%, *P* < 0.05), whereas the dilation induced by ACh (10 µM) remained unaffected (Con, 63.8 ± 7.8; DPSX, 51.5 ± 7.1%; *P* = ns). Dilations in response to muscle contraction were mostly unaffected in the presence of DPSX (Fig. 2a). Only at a stimulation frequency of 100 Hz for a duration of 60 s the dilation tended to be smaller at the end of the stimulation period during DPSX (Con, 83.0 ± 3.9; DPSX, 65.4 ± 7.3; *P* = 0.10).

Application of LN and Indo reduced arteriolar diameters from 9.5 ± 1.3 to 5.6 ± 0.8 µm (*P* < 0.05) in this subgroup (*n* = 8). Dilations in response to 10 µM acetylcholine were not altered (Con, 78.4 ± 5.2; LN + Indo, 76.2 ± 5.5%; *P* = ns), whereas the dilations induced by 10 µM adenosine tended to be smaller (Con, 58.2 ± 10.0; LN + Indo, 40.9 ± 8.1%; *P* = 0.08). The dilations induced by motor nerve stimulation with 100 Hz were not modulated in the presence of LN and Indo (Fig. 2b).

In the third subgroup, these treatments were applied in combination (*n* = 24). DPSX, LN, and Indo reduced resting arteriolar diameter from 8.1 ± 0.7 to 4.4 ± 0.6 µm (*P* < 0.05). The dilation in response to adenosine (10 µM) was strongly reduced compared to the untreated control condition (from 61.4 ± 5.0 to 18.0 ± 4.1%; *P* < 0.05), while the dilation induced by ACh was not significantly altered (10 µM: from 69.5 ± 4.4 to 59.0 ± 7.3%; *P* = ns). Dilations upon initiation of skeletal muscle contraction using 100 Hz stimulation were reduced by DPSX, LN, and Indo (Fig. 2c). This reduction was evident at the end of the stimulation if it lasted for 15, 30, or 60 s, but not for the brief stimulation period of 1 s. Accordingly, a significant reduction was also observed during the stimulation if the stimulation lasted for 30 or 60 s but not if it lasted only for 15 s. Thus, the reduction was related to the dilatory amplitude which increased with stimulation duration. In contrast, the dilation upon 1-s stimulation was completely undisturbed (Fig. 2c).

### K_ATP_ channel blockade in combination with inhibition of NO synthase and cyclooxygenase nearly abrogates active hyperemia

**Fig. 3:**
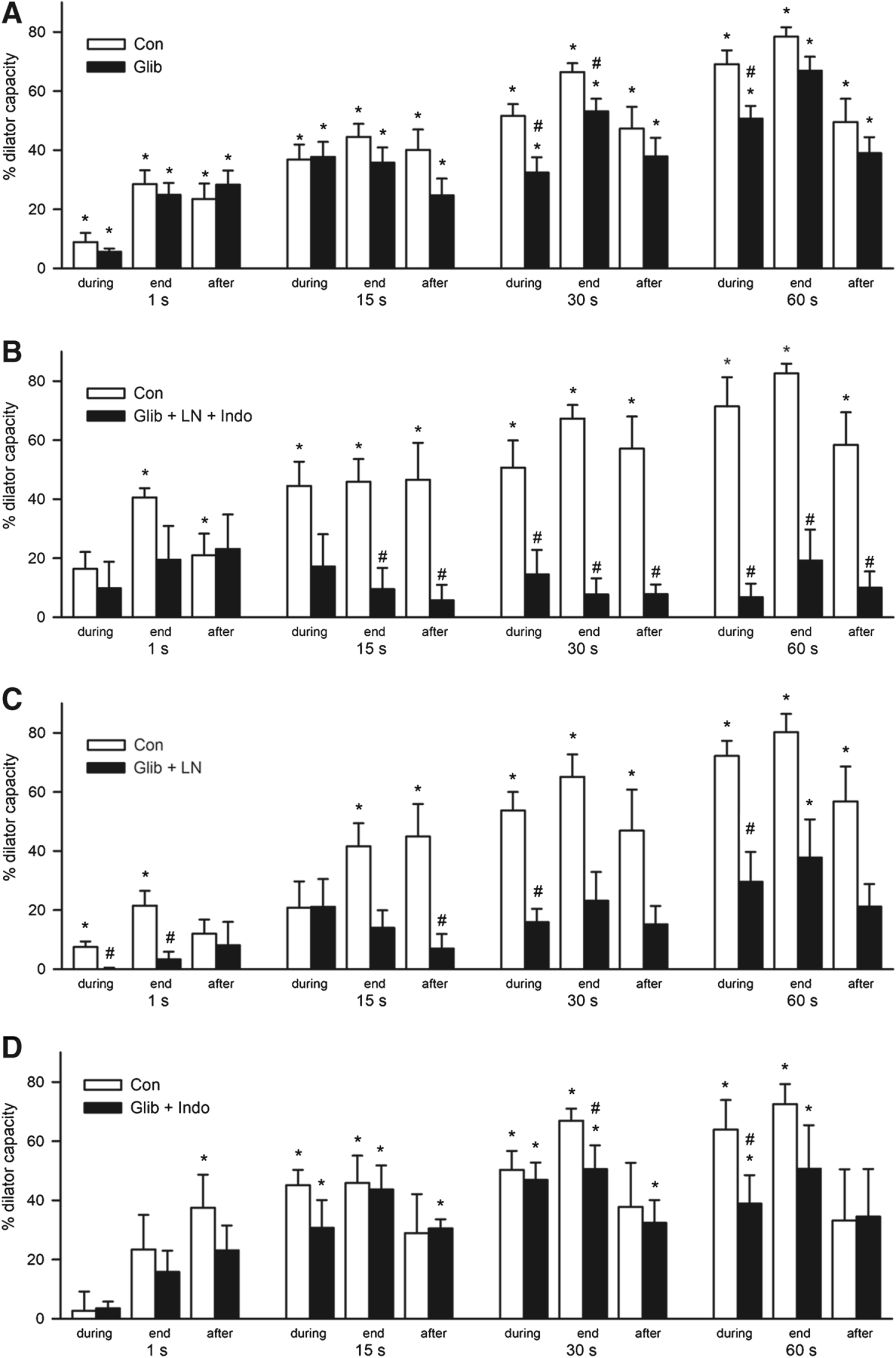
K_ATP_ channel blockade in combination with inhibition of NO synthase and cyclooxygenase nearly abrogates active hyperemia. Arteriolar dilations during, at the end of, and after stimulation of skeletal muscle contraction at 100 Hz are depicted for the different stimulation periods (for definition of time points, see legend of Fig. 2). Treatments are shown in black bars; the respective control values obtained in the exact same arterioles are depicted in white bars. **A** Application of the K_ATP_ channel blocker glibenclamide (10 µM, Glib, *n* = 15) only moderately reduced the arteriolar dilations at longer stimulation periods. **B** Additional inhibition of NO synthase and cyclooxygenase (LN, Indo; 30 and 3 µM, *n* = 5) strongly reduced the dilations. At all time points for all stimulation periods, a significant diameter change was not observed and the dilations were attenuated compared to controls. **C** If only NO synthase (LN, 30 µM) was inhibited combined with Glib (*n* = 5), the inhibitory effect on arteriolar dilations was likewise observed except for the longest stimulation period. However, the dilation during 60-s stimulation was also significantly reduced compared to control. **D** In contrast, cyclooxygenase inhibition (Indo, 3 µM) combined with Glib exerted only a limited inhibitory effect that resembled the treatment with glibenclamide alone (shown in (**A**)). Dilation is given as percent of dilator capacity, *P < 0.05 vs diameter before stimulation. #*P* < 0.05 vs dilation of respective control; diameter values are given in Table 1

In a different set of experiments the K_ATP_ channel blocker glibenclamide (Glib, 10 µM) was applied followed by either LN or Indo or a combination of both. Application of Glib reduced resting arteriolar diameters from 6.7 ± 0.5 to 4.8 ± 0.5 µm (*n* = 15, *P* < 0.05). The dilation in response to adenosine (10 µM) was reduced from 49.3 ± 5.3 to 14.7 ± 6.2% (*P* < 0.05), and the ACh-induced dilation (10 µM) was also mildly attenuated (from 66.0 ± 5.5 to 42.9 ± 8.1%, *P* < 0.05). Compared to the respective control condition, the dilations induced by skeletal muscle contraction were moderately reduced (Fig. 3a). Arteriolar dilation was attenuated during and at the end of the stimulation period of 30 s and also during the 60-s stimulation period. At the end of the 60-s stimulation, a tendency for a reduction was observed (78.4 ± 3.2 vs 66.9 ± 4.7%, *P* = 0.10). This was similarly found at the end of the 15-s stimulation period (44.5 ± 4.4 vs 35.8 ± 5.1%, *P* = 0.07). However, dilations during 1-s stimulation remained unaffected.

In a subgroup of experiments, LN and Indo were added in addition to Glib (*n* = 5). This treatment reduced arteriolar resting diameter from 5.6 ± 1.2 to 1.5 ± 0.4 µm (*P* < 0.05). The adenosine-induced dilation was significantly reduced (10 µM: from 55.0 ± 9.1 to 20.1 ± 5.5%, *P* < 0.05), whereas the ACh induced was not significantly affected (10 µM: 68.8 ± 5.2 to 49.5 ± 18.2%, *P* = ns, control vs Glib + LN + Indo). The dilations in response to skeletal muscle contraction (100 Hz) were nearly abrogated. In contrast to the control condition, a significant diameter change during, at the end of, or after skeletal muscle contraction was not observed at all stimulation periods in the presence of Glib + LN + Indo (Table 1). The calculated dilations were significantly reduced compared to control dilations for the stimulation periods of 15, 30, and 60 s (Fig. 3b).

In two different subgroups, LN or Indo was combined separately with Glib in order to analyze the importance of NO synthase and cyclooxygenase activity in this setting. Both treatments reduced arteriolar resting diameters (Glib + LN, from 6.3 ± 0.4 to 2.5 ± 0.4 µm, *P* < 0.05; Glib + Indo, from 8.1 ± 0.8 to 4.4 ± 0.9 µm, *P* < 0.05; each *n* = 5). The dilations induced by adenosine (10 µM) were again reduced (Glib + LN, from 53.9 ± 7.8 to 21.0 ± 5.4%, *P* < 0.05; Glib + Indo, from 43.8 ± 12.1 to 7.9 ± 2.3%, *P* < 0.05), and dilations to ACh (10 µM) were mildly attenuated (Glib + LN, from 68.4 ± 7.0 to 48.3 ± 20.2%, *P* = ns; Glib + Indo, from 60.7 ± 15.2 to 42.0 ± 11.9%, *P* < 0.05). In preparations treated with Glib and LN, skeletal muscle contraction did induce significant diameter changes only upon a stimulation period of 60 s, but not, in contrast to the respective control condition, upon stimulation periods of 1, 15, or 30 s (Fig. 3c, Table 1). In contrast, significant diameter changes were found in the presence of Glib and Indo at the end of the 15, 30, and 60-s stimulation periods (Fig. 3d). Only in some instances a significant reduction of the dilation was observed in this group (at the end of 30-s stimulation and during 60-s stimulation) which were very similar to the differences found in preparations treated only with Glib.

## Discussion

This study demonstrates that skeletal muscle contraction induces a robust dilation in the murine microcirculation at the level of intramuscular arterioles specifically at higher stimulation frequencies (100 Hz) that induce tetanic contractions. These dilations require muscular contractions and are not mediated by the release of acetylcholine from the neuromuscular synapse. The exclusive blockade of adenosine receptors or K_ATP_ channels or the combined blockade of NO synthase and cyclooxygenase alone exerts very little if any attenuation of the dilation. In contrast, the inhibition of NO synthase and cyclooxygenase combined with the blockade of either adenosine receptors or K_ATP_ channels reduced the dilation preferentially for longer stimulation periods. In this combination, blockade of K_ATP_-channels was more efficient than adenosine receptor blockade in reducing the dilation. This suggests that, in addition to adenosine, other mediators or mechanisms contribute to exercise induced hyperemia in the murine skeletal muscle microcirculation by activation of K_ATP_ channels.

Our protocol of electrical stimulation to induce skeletal muscle contraction entailed a stimulation period of 0.5 s followed by a break of 2.5 s in order to achieve full visibility of the arteriole under study during the whole stimulation. As expected, the amplitude of the arteriolar dilation increased with prolonged stimulation at both frequencies studied. However, the slope was much steeper, and the resulting dilation was considerably larger, more robust, and highly reproducible if tetanic contractions (100 Hz) were elicited. Therefore, the effect of inhibitors was examined systematically using this stimulation frequency. The slope of the dilation flattened with longer stimulation durations as was reported previously [1], and, most interestingly, any further progress of the dilation was immediately (within seconds) interrupted after cessation of the electrical stimulation. This suggests that the dilator mechanisms are rapidly turned on and, conversely, any further dilatory stimulation is also immediately switched off. This implies that the mechanisms involved are not only promptly active and efficient as highlighted also before [24, 43] but it also suggests that the dilatory mechanisms are rapidly inactivated and exert their effect upon activation/release only for a short time in the range of seconds. Depending on the duration of the electrical stimulation (and the dilator amplitude achieved), the arterioles gradually return to their initial resting diameters. The return close to the initial diameter took about four to five times as long as the stimulation period had lasted with the exception of the response upon the brief stimulation period of 1 s after which the return to baseline took longer than 5 s. Overall, this indicates that the initiation of the dilation is a faster process than the return to baseline. This difference may well be related to an oxygen deficiency that has accumulated during skeletal muscle contraction.

Acetylcholine spillover from neuromuscular synapses reportedly contributes to the dilation by possibly stimulating endothelial cells to release dilatory mediators [47]. In addition to NO, acetylcholine initiates specifically in mice an EDH mechanism to induce arteriolar dilation [45, 49]. This proposed action of acetylcholine in conjunction with neuromuscular activation may also be accountable for the otherwise elusive function of muscarinic receptors in endothelial cells [39]. However, we did not find any evidence for this hypothesis because the blockade of nicotinergic receptors by pancuronium which completely prevented muscular contraction (as observed in the microcirculation) also completely abolished arteriolar dilations and left the dilation upon externally applied acetylcholine intact. It is very likely to assume that the electrical activation of the motor nerve still released acetylcholine at the synapse during pancuronium application, yet the dilation was abolished which indicates that skeletal muscle contraction is indeed required to initiate a hyperemic response.

It has been suggested that the search for a single key dilator mechanism is futile and the nature of the vasoregulatory system specifically in the context of serving tissue needs is one of redundancy [20, 31, 43]. This may be achieved by different mechanisms activated and the release of different substances which is initiated by the tissue that is in need of more oxygen delivery. The present results in murine skeletal muscle support this concept since sole blockade of adenosine receptors or the blockade of the endothelial mediators NO and prostaglandins did not attenuate the dilator response. An efficient blockade of adenosine receptors by DPSX was verified by the near complete elimination of the dilation in response to externally applied adenosine, which induced a dilation that was of a comparable magnitude as the dilation in response to electrical activation of the skeletal muscle. It should be noted that DPSX did not modulate resting arteriolar diameter indicating that adenosine is of no importance in the control of basal tone. The inhibition of NO synthase and cyclooxygenase reduced the resting diameter suggesting an efficient blockade and verifying an effect of NO in the control of basal tone as demonstrated previously [49]. A minor tendency to reduce the adenosine dilation suggests that this dilation may be partially mediated by these endothelial mediators in mice while in other species adenosine-induced dilations are reported to be mediated substantially by NO release [5, 13, 27]. Other studies suggested that adenosine acts through signaling via endothelial cells [10] although prostaglandins and NO were not involved [29]. Importantly, the combined blockade of adenosine receptors and NO/prostaglandin synthesis attenuated the dilation in response to electrically stimulated muscle contraction. The reduction was observed preferentially during longer stimulation periods (> 10 s) but not at all during very brief stimulatory periods. This demonstrates that endothelial NO and prostaglandins together with adenosine are indispensable for a fully intact hyperemic dilation of intramuscular arterioles in mice. Adenosine and NO/prostaglandins may replace each other and are redundant mechanisms, but blockade of both pathways mitigated the dilation by roughly one third. This also indicates that they do act independently rather than in synergy and are not dependent on each other in the sense that NO (or prostaglandins) mediate the dilation upon adenosine.

In the coronary circulation, blockade of K_ATP_ channels reduced blood flow [9] and also attenuated active hyperemic dilation in skeletal muscle in hamsters [3]. In the present experiments, glibenclamide, a blocker of K_ATP_ channels, reduced arteriolar resting diameters and substantially attenuated the dilation upon adenosine. This suggests that these channels contribute to the control of resting diameters and mediate a large fraction of the adenosine-induced dilation in murine arterioles. Moreover, this indicates that the concentration used (10 µM) effectively blocks K_ATP_ channels in this preparation. Dilations induced by electrical stimulation were moderately attenuated at longer stimulation periods (> 15 s) in the presence of glibenclamide. Importantly, if additionally the release of the endothelial mediators NO and prostaglandins was inhibited, the dilation in response to electrical stimulation of the skeletal muscle was severely impaired. In fact, a significant diameter change was not found anymore in this small sized experimental subgroup (*n* = 5). A tendency for a dilation was left at these conditions and a larger experimental size may have demonstrated a remaining dilation. This suggests that these dilator mechanisms are, as observed before, redundant and the lack of an activation of K_ATP_ channels can be rescued by NO and prostaglandin release. Of these two endothelial mediators, NO is crucial, because the effect of a combination of glibenclamide and an inhibitor of NO-synthase reduced the dilations in a comparable manner, whereas the combined addition of cyclooxygenase inhibition and glibenclamide did not strengthen the inhibitory effect of glibenclamide alone. These experiments suggest that K_ATP_ channels and NO are important mediators of arteriolar dilations induced by skeletal muscle contraction in mice. They are able to replace each other to a large extent, and cyclooxygenase products may support the dilation to a very minor degree. The arteriolar dilation in response to direct muscle fiber stimulation in hamsters also involved K_ATP_ channels and NO [3, 8].

K_ATP_ channels are reported to be activated by adenosine in other species [11, 12]. We also demonstrated previously that K_ATP_ channels are required for adenosine-induced dilations in murine arterioles (most likely in smooth muscle cells) [7] as was verified in the present experiments. Therefore, we did not examine the combined blockade of K_ATP_ channels and adenosine receptors in hyperemic responses. In light of the larger inhibitory effect of glibenclamide compared to adenosine receptor blockade, one may speculate that other mediators also exert a dilatory effect through activation of K_ATP_ channels during exercise hyperemia.

In conclusion, we demonstrated that electrical stimulation of skeletal muscle induces robust and rapid dilations of intramuscular arterioles that are mediated by adenosine acting via opening of K_ATP_ channels and NO. These mechanisms are redundant and are only uncovered by their simultaneous inhibition. Prostaglandins may add with a minor importance to the dilation in conjunction with other players which act through opening of Ca^2+^-dependent K^+^ channels as demonstrated before [26]. Most interestingly, the contribution of K^+^ channels and consequent hyperpolarization of the cells of the vessel wall sets the stage for ascending or conducting dilations that are crucial for an overall response along the arteriolar network.

## Data Availability

Not applicable.
